# Glanzmann thrombasthenia presenting with upper gastrointestinal bleeding: a case series and review of the literature

**DOI:** 10.3389/fmed.2026.1762228

**Published:** 2026-02-16

**Authors:** Feras Al-Fararjeh, Alaa Alshorman, Salem Hyari, Manar Abu Awwad, Hussam I. A. Alzeerelhouseini, Renad Alawamleh, Eman Abdelghani, Sally Al-Aqrabwi, Abdalla Awidi

**Affiliations:** 1 Division of Hematology and Medical Oncology, Medical School, The University of Jordan, Amman, Jordan; 2 Al-Basheer Hospital, Ministry of Health, Amman, Jordan; 3 Internal Medicine Resident, Jordan University Hospital, The University of Jordan, Amman, Jordan; 4 Indiana Hemophilia and Thrombosis Center, Innovative Hematology Inc, Indiana, IN, United States; 5 Cell Therapy Center, The University of Jordan, Amman, Jordan

**Keywords:** gastrointestinal hemorrhage, Glanzmann thrombasthenia, GPIIb/IIIa receptor, inherited platelet disorders, recombinant factor VIIa

## Abstract

**Background:**

Glanzmann thrombasthenia (GT) is a rare autosomal recessive bleeding disorder characterized by a defect in the platelet integrin αIIbβ3. While mucocutaneous bleeding is typical, gastrointestinal (GI) bleeding is not uncommon and can be a potentially severe complication.

**Materials and methods:**

A total of six patients, including four female and two male individuals with type I GT, presented with massive upper GI bleeding of unknown cause. Treatment was selected based on the severity of each case and included a combination of recombinant activated factor VII (rFVIIa), tranexamic acid, endoscopic intervention, and supportive care. Genetic studies were performed for each patient.

**Results:**

The endoscopic findings were heterogeneous, varying from normal mucosa to visible vessels, polyps, and arteriovenous malformations. All cases were managed successfully.

**Discussion:**

The unpredictable nature of GI bleeding in patients with GT highlights the need for careful clinical assessment and a multidisciplinary management approach. Bleeding control was achieved through the use of antifibrinolytic agents and recombinant factor VIIa, which also reduces the risk of platelet alloimmunization. A possible link between certain *ITGB3* variants and severe bleeding suggests phenotypic variability.

**Conclusion:**

This case series highlights the role of careful management strategies in reducing morbidity among patients with GT and adds to the limited literature on gastrointestinal bleeding in this population.

## 1 Introduction

Glanzmann thrombasthenia (GT) is a rare autosomal recessive bleeding disorder arising from quantitative or qualitative defects in the platelet glycoprotein (GP) IIb/IIIa complex (αIIbβ3 integrin). This receptor is essential for fibrinogen binding and platelet aggregation, and its dysfunction leads to impaired clot formation (1). First described in 1918 by Glanzmann, GT is considered rare in most populations but has higher prevalence in regions with consanguineous marriages, such as Jordan (2–5).

The clinical hallmark of GT is lifelong mucocutaneous bleeding, including epistaxis, gingival hemorrhage, and menorrhagia (6). Gastrointestinal (GI) bleeding is not an uncommon manifestation, reported in 10–20% of patients, and it can be severe, life-threatening, and unpredictable (7). GT diagnosis is confirmed by demonstrating absent or impaired platelet aggregation in response to ADP, collagen, and epinephrine, with normal ristocetin-induced agglutination (7–9). Flow cytometry shows absent or decreased CD41 (GPIIb) and CD61 (GPIIIa) expression, and genetic testing is crucial for definitive subtyping and counseling (7, 8).

GT is classified into three types: Type I (severe, <5% GPIIb/IIIa), Type II (moderate, 5–20% GPIIb/IIIa), and Type III (>20% to normal levels of dysfunctional protein) (10). Management of bleeding episodes is supportive, involving local measures such as cauterization, antifibrinolytics (e.g., tranexamic acid), and systemic therapies such as platelet transfusions or recombinant activated factor VII (rFVIIa) (11, 12). A significant long-term challenge is the development of antiplatelet antibodies, leading to platelet refractoriness (6).

We present a case series of six patients with type I GT who experienced upper GI bleeding. This report aims to clarify the heterogeneous presentation and management challenges of this complication, supplemented by a comprehensive review of the literature. All patients showed complete resolution of bleeding after supportive treatment.

## 2 Materials and methods

A total of six individuals with GT experienced upper GI bleeding at the University of Jordan Hospital between 2010 and 2025. The individuals were invited to participate in this research protocol after the Institutional Ethics Committee had approved it (IRB/4/2017-17). Written informed consent was obtained from the patients. Data were obtained from medical records and confirmed by the patients.

Platelet aggregation testing was performed using multiple electrode aggregometry with the multiple platelet function analyzer (Multiplate^®^ Analyzer, Roche Diagnostics, Germany). Hirudin blood tubes were used, and the following agonists were applied: ADP 6.4 μM (ADPtest), collagen 3.2 μg/mL (COLtest), ristocetin 1.5 mg/mL (RISTO high), and 0.5 mg/mL (RISTO low).

Flow cytometry analysis was performed on whole blood to assess platelet surface glycoprotein expression (CD41 and CD61). Molecular studies included Sanger sequencing of the coding regions of the ITGB3 and ITGA2B genes. DNA was extracted from peripheral blood using a commercial kit (Qiagen MinElute, Germany). Samples were analyzed on an ABI 3500 Genetic Analyzer (Applied Biosystems, United States). Sequencing files were aligned to detect point mutations using Chromas Pro (Technelysium, Australia) against the reference transcript ENST00000559488.7. DNA variants were classified using an automated variant classification tool (https://franklin.genoox.com, accessed on 23 May 2025). Novel mutations were confirmed by their absence in any previously published studies.

## 3 Results

We describe six patients with clinically confirmed type I GT who presented with upper GI bleeding. All patients had normal platelet counts and morphology, prolonged bleeding time (>13 min), poor clot retraction, and absent platelet aggregation in response to ADP and collagen, with normal aggregation in response to ristocetin. The flow cytometry findings were consistent with αIIbβ3 deficiency. Prior to the GI bleeding episodes, all patients had a history of recurrent mucocutaneous bleeding at various sites other than the GI tract and had received red blood cell and platelet transfusions. Key clinical characteristics and genetic results are summarized in Table 1.

**Table 1:** Summary of endoscopic findings and management of gastrointestinal bleeding in the present case series.

Case	Age/Sex	Diagnosis age	Mutation	Presentation	Hb (g/dL)	Findings	Management
1	53/F	First year	c.1723 T > C Pathogenic	ME/HE	4.6	Upper gastrointestinal bleeding	3 units BT, rFVIIa, TA
2	26/F	At birth	c.1723 T > C Pathogenic	ME/HE	5.9	Gastric hemorrhagic spots	2 units BT, rFVIIa, TA
3	47/M	First year	c.1723 T > C Pathogenic	ME/HE	6.0	Gastritis/duodenal ulcer	4 units BT, TA, AH
4	50/F	Two years	Unknown	ME, NSAID	5.6	Gastric bleeding polyp	4 units BT, PP, rFVIIa, TA
5	59/M	Three years	Unknown	ME, Diz	6.7	Duodenal arteriovenous malformation	APC, rFVIIa, PPI
6	46/F	At birth	c.783 del G Likely pathogenic^a^	ME	10.0	Gastric and colonic polyps	BT, rfVIIa, L.inj

a Novel mutation.

ME, melena; HE, hematemesis; Diz, dizziness; BT, blood transfusions; rfVIIa, recombinant factor VIIa; TA, tranexamic acid; AH, absorbable hemostat; PP, polypectomy; APC, argon plasma coagulation; PPI, proton pump inhibitor; L.inj, lanreotide injection.

### 3.1 Case 1

A 53-year-old female individual with a confirmed homozygous mutation, c.1723 T > C (p.Cys575Arg), in the *ITGB3* gene. She presented six times over 10 months with melena and hematemesis, with hemoglobin (Hb) dropping to 4.6 g/dL during the last visit to the emergency room. Endoscopy revealed multiple bleeding vessels in the duodenum and stomach. Bleeding was controlled during each episode using rFVIIa (administered at 0 h, 2 h, 8 h, and 24 h for 3 days) and intravenous tranexamic acid (1 g every 6 h for 3 days).

### 3.2 Case 2

A 26-year-old female individual with a family history of GT and a confirmed homozygous mutation, c.1723 T > C (p.Cys575Arg), in the *ITGB3* gene. She presented with continuous hematemesis and melena for 3 days, with Hb dropping to 5.9 g/dL. Endoscopy showed hemorrhagic spots in the gastric fundus, antrum, and body, without evidence of ulcers. She was treated with omeprazole (40 mg IV) and successfully managed with rFVIIa (administered at 0 h, 2 h, 8 h, and 24 h for 3 days) and tranexamic acid (1 g every 6 h for 3 days).

### 3.3 Case 3

A 47-year-old male individual with a homozygous mutation, c.1723 T > C (p.Cys575Arg), in the *ITGB3* gene. He had two episodes of upper GI bleeding. In 2010, he presented with hematemesis (Hb 6 g/dL); endoscopy showed gastritis and erosions. He was treated with blood transfusion and omeprazole and was advised to avoid NSAIDs. In the second episode in 2021, he presented with melena (Hb 7.2 g/dL); upper GI endoscopy identified a bleeding point in the duodenum, which was controlled with an absorbable hemostat and tranexamic acid (1 g every 6 h). He was discharged after 24 h.

### 3.4 Case 4

A 50-year-old female individual with a family history of GT and frequent NSAID use. She presented with melena for 3 days; her Hb reached 5.6 g/dL. Endoscopy revealed a bleeding gastric polyp, which was removed via polypectomy. She received a blood transfusion, omeprazole, rFVIIa (administered at 0 h, 2 h, 8 h, and 24 h for 3 days), tranexamic acid (1 g every 6 h), and supportive care. After 5 days, an upper GI endoscopy was repeated and showed no active bleeding.

### 3.5 Case 5

A 59-year-old male individual presented with melena for 4 days and dizziness, with Hb dropping to 6.7 g/dL. Upper endoscopy identified a bleeding arteriovenous malformation in the duodenum, which was treated with argon plasma coagulation. He received rFVIIa (administered at 0 h, 2 h, 8 h, and 24 h for 3 days) and supportive care.

### 3.6 Case 6

A 46-year-old female individual with no family history of GT, found to carry a homozygous single-nucleotide deletion, c.783 del G (p.Lys261fs), in the *ITGB3* gene. She presented with melena in 2022, and her Hb was 7 g/dL. Treatment included blood transfusion and tranexamic acid (1 g every 6 h for 3 days). The patient refused upper endoscopy. Her symptoms persisted, and at the beginning of 2025, she underwent upper and lower endoscopy. Upper endoscopy revealed large fundic gland-appearing polyps with active oozing, and the lower endoscopy revealed three sessile polyps in the transverse colon, measuring 6–8 mm. She received rFVIIa following the same dosing schedule as the other cases, along with blood transfusion, lanreotide injections, and supportive care. Recently, a laparoscopic proximal subtotal gastrectomy with double-tract reconstruction was performed, and since then, she reported no further episodes of melena.

## 4 Discussion and review of the literature

Our case series illustrates the significant challenge of upper GI bleeding in GT, a complication that, while uncommon, can dominate the clinical course and lead to life-threatening anemia (13). The heterogeneity of our cases reflects the broader literature, where GI bleeding in GT can arise from diverse etiologies, including idiopathic mucosal oozing, discrete lesions, or vascular malformations (14–16).

A pivotal discussion point is the occurrence of GI bleeding in which the source and localization of bleeding could not be identified endoscopically, as observed in case 2. This phenomenon has been reported before (7, 17) and suggests diffuse vascular fragility inherent to the GT phenotype. The absence of the αIIbβ3 integrin may impair the integrity of the subepithelial vascular network or the initial platelet plug required to seal microscopic injuries, leading to significant bleeding from lesions that are invisible on conventional endoscopy (18).

Conversely, our case series also includes patients with identifiable structural causes. Case 4 had a bleeding polyp, exacerbated by NSAID use, highlighting the critical importance of avoiding antiplatelet agents in GT (19). Case 5 had a duodenal arteriovenous malformation (DAVM), a known association in GT (16, 20). The co-occurrence of a bleeding diathesis and vascular anomalies creates a perfect storm for severe hemorrhage. Other reported lesions include *H. pylori*-associated ulcers (21) and multiple gastric polyps requiring gastrectomy (22). This spectrum of findings underscores the need for a thorough endoscopic evaluation in any GT patient with GI bleeding, even when the mucosa appears grossly normal (20, 23).

The management of these episodes requires a multidisciplinary approach. Antifibrinolytic therapy with tranexamic acid is a cornerstone, reducing fibrinolytic activity at the mucosal surface (23). For major bleeding in previously transfused patients, rFVIIa has become an important agent for the treatment because the devolving of anti-platelets antibodies. It promotes thrombin generation on the surface of activated platelets independently of the IIb/IIIa receptor, effectively “bypassing” the defect (24). The high success rate (up to 91%) of rFVIIa, often combined with antifibrinolytics, is well documented in the GT registry (24). Its use is particularly advantageous in reducing the risk of alloimmunization associated with platelet transfusions, which is a well-recognized long-term complication in patients requiring repeated transfusions (6). The efficacy of this strategy is demonstrated in our series, where rFVIIa was used successfully in five of the six cases.

For refractory bleeding due to vascular anomalies, as described in the literature, advanced pharmacological agents include octreotide (to reduce splanchnic blood flow), thalidomide, and bevacizumab (anti-VEGF therapy) (16, 25). Hormonal therapy has also been used to reduce transfusion requirements in patients with diffuse vascular abnormalities (16). Although not required in our cases, these options represent important tools for complex scenarios. Ultimately, hematopoietic stem cell transplantation remains the only curative option for severe GT (26).

Genotype–phenotype correlations in patients suggest a possible link between specific *ITGB3* variants and the severity of gastrointestinal bleeding in GT. However, the response to rFVIIa varies, showing differences in bleeding severity even among patients with the same genotype. Cases 1, 2, and 3, which experienced significant upper GI bleeding, carried the pathogenic missense mutation *ITGB3* c.1723 T > C, a variant known to impair αIIbβ3 function (27). Case 6 experienced severe and progressive clinical GI bleeding and carried a likely pathogenic frameshift variant, *ITGB3* c.783delG, which is expected to result in loss of protein expression. This strongly suggests that the frameshift mutation leads to more severe and complex GI bleedin*g* compared to the missense mutation group. In contrast, two patients (Cases 4 and 5) who experienced GI bleeding showed no identifiable pathogenic or likely pathogenic point mutations in *ITGB3*, making it difficult to establish a genetic basis for their phenotype. Although the small sample size limits this correlation, these findings suggest that while certain *ITGB3* mutations may contribute to a more severe bleeding profile, GI bleeding can still occur in the absence of detectable pathogenic variants.

Our findings, supported by evidence from the literature (Table 2), reinforce key management principles: Maintaining a high index of suspicion for GI bleeding, performing aggressive endoscopic localization and intervention when possible, and prioritizing rFVIIa over platelet transfusions to reduce the risk of alloimmunization. Eradication of *H. pylori* and strict avoidance of NSAIDs are essential prophylactic strategies.

**Table 2:** Summary of the literature review of GT patients with upper GI bleeding.

Ref	*n*	Age/Sex	GT type	Presentation	Management	Result
Duncan et al. (7)	1	43/M	I	ME, w/o.L	PT, BT, PPI	Resolved
Salih et al. (17)	1	17/M	N/A	ME, HE, EP, w/o.L	PT, PPI	Resolved
Calabrese et al. (21)	1	52/F	II	*ME, HE, DU*	BT, PT, PE, HPer*, HT,* TA	Resolved
Bakdash et al. (22)	1	48/F	I	ME, AN, GP, FOB	BT, PPI, Ran, HC, APC, PG, rFVIIa	Resolved
Coppola et al. (16)	1	75/F	I	ME, AN, MVA, w/L	BT, AFC, HT	Reduced bleeding
Tarawah and Tarawah (20)	8	Diverse	I	ME, AD	rFVIIa, PT, BT	One died

*n*, number of cases; ME, melena; w/o.L, without lesion; PT, platelet transfusion; BT, blood transfusion; PPI, proton pump inhibitor; HE, hematemesis; DU, duodenal ulcer; HPer, *H. Pylori* eradication; HT, hormone therapy; TA, tranexamic acid; AN, anemia; GP, gastric polyps; FOB, fecal occult blood; Ran, ranitidine; HC, hemostasis clip; APC, argon plasma coagulation; PG, partial gastrectomy; MVA, mucosal vascular abnormalities; w/L, with lesion; AD, angiodysplasia; EP, epigastric pain; PE, plasma expander; AFC, antifibrinolytics.

## 5 Conclusion

Upper gastrointestinal bleeding is a severe and unpredictable complication of Glanzmann thrombasthenia. It can occur in the absence of visible mucosal lesions or in association with specific pathologies such as ulcers, polyps, or vascular malformations. Maintaining a high index of suspicion and performing prompt endoscopic evaluation are crucial. Management should be multifaceted, emphasizing the use of antifibrinolytics and rFVIIa as first-line systemic therapies in previously transfused patients to control bleeding effectively while minimizing the risk of platelet alloimmunization. For complex cases with vascular anomalies, advanced pharmacological agents provide additional options. Heightened awareness of this complication and a structured management approach are essential to mitigate the significant morbidity associated with GI bleeding in GT.

## Data Availability

The datasets analyzed during the current study are not publicly available due to ethical, legal, and privacy restrictions involving human participants and genetic data, in accordance with institutional ethics approval. Requests to access the datasets should be directed to the corresponding author.
